# Cord Serum Calcitriol Inversely Correlates with Maternal Blood Pressure in Urinary Tract Infection-Affected Pregnancies: Sex-Dependent Immune Implications

**DOI:** 10.3390/nu13093114

**Published:** 2021-09-04

**Authors:** Andrea Olmos-Ortiz, Alberto Olivares-Huerta, Janice García-Quiroz, Euclides Avila, Ali Halhali, Braulio Quesada-Reyna, Fernando Larrea, Verónica Zaga-Clavellina, Lorenza Díaz

**Affiliations:** 1 Departamento de Biología de la Reproducción “Dr. Carlos Gual Castro”, Instituto Nacional de Ciencias Médicas y Nutrición Salvador Zubirán, Av. Vasco de Quiroga No. 15, Belisario Domínguez Sección XVI, Tlalpan, Ciudad de Mexico 14080, Mexico; nut.aolmos@gmail.com (A.O.-O.); dr.alberto.olivares@gmail.com (A.O.-H.); janice.garciaq@incmnsz.mx (J.G.-Q.); euclides.avilac@incmnsz.mx (E.A.); ali.halhalib@incmnsz.mx (A.H.); fernando.larreag@incmnsz.mx (F.L.); 2 Departamento de Inmunobioquímica, Instituto Nacional de Perinatología Isidro Espinosa de los Reyes, Montes Urales 800, Lomas-Virreyes, Lomas de Chapultepec IV Sección, Miguel Hidalgo, Ciudad de Mexico 11000, Mexico; 3 División de Obstetricia, UMAE Hospital de Gineco-Obstetricia No. 4 “Luis Castelazo Ayala”, IMSS, Rio de la Magdalena 289, Tizapán San Ángel, Progreso Tizapán, Álvaro Obregón, Ciudad de Mexico 01090, Mexico; braulio.quesada@imss.gob.mx; 4 Departamento de Fisiología y Desarrollo Celular, Instituto Nacional de Perinatología Isidro Espinosa de los Reyes, Montes Urales 800, Lomas-Virreyes, Lomas de Chapultepec IV Sección, Miguel Hidalgo, Ciudad de Mexico 11000, Mexico

**Keywords:** urinary tract infection, vitamin D, blood pressure, calcitriol, pregnancy

## Abstract

Urinary tract infections (UTI) during pregnancy are frequently associated with hypertensive disorders, increasing the risk of perinatal morbidity. Calcitriol, vitamin D_3_’s most active metabolite, has been involved in blood pressure regulation and prevention of UTIs, partially through modulating vasoactive peptides and antimicrobial peptides, like cathelicidin. However, nothing is known regarding the interplay between placental calcitriol, cathelicidin, and maternal blood pressure in UTI-complicated pregnancies. Here, we analyzed the correlation between these parameters in pregnant women with UTI and with normal pregnancy (NP). Umbilical venous serum calcitriol and its precursor calcidiol were significantly elevated in UTI. Regardless of newborn’s sex, we found strong negative correlations between calcitriol and maternal systolic and diastolic blood pressure in the UTI cohort (*p* < 0.002). In NP, this relationship was observed only in female-carrying mothers. UTI-female placentas showed higher expression of cathelicidin and CYP27B1, the calcitriol activating-enzyme, compared to male and NP samples. Accordingly, cord-serum calcitriol from UTI-female neonates negatively correlated with maternal bacteriuria. Cathelicidin gene expression positively correlated with gestational age in UTI and with newborn anthropometric parameters. Our results suggest that vitamin D deficiency might predispose to maternal cardiovascular risk and perinatal infections especially in male-carrying pregnancies, probably due to lower placental CYP27B1 and cathelicidin expression.

## 1. Introduction

During pregnancy, maternal cardiovascular and renal physiology undergo profound changes to favor adequate perfusion of the uteroplacental unit. These changes include increased cardiac output, stroke volume, blood volume, and heart rate as well as the increment in the circulation of molecules that rise blood pressure and body-fluid volume, such as renin and angiotensin (ANG) II, which are components of the renin-angiotensin system (RAS) [1,2]. Despite these changes and RAS activation throughout normal pregnancies, systolic and diastolic blood pressure (SBP, DBP) are significantly decreased compared to non-pregnant values. At the same time, peripheral vascular resistance is reduced [3,4]. The latter is partially explained by the concomitant increase in peripheral vasodilator levels in maternal serum, such as prostacyclin, progesterone, and ANG 1–7 [1,4,5,6,7]. The placenta is a major site of production of these and other vasoactive factors, contributing in this manner to systemic and uteroplacental hemodynamics. Disruption or dysregulation of the mechanisms involved in these processes may result in gestational hypertensive disorders, including preeclampsia, as shown when an excessive secretion of renin from placental origin is released into the maternal circulation [8,9]. Calcitriol is another important placental factor that spills into the maternal circulation and modulates feto-maternal physiology [10,11,12]. Calcitriol is the hormonal form and most active metabolite of vitamin D_3_ (VD), produced from its precursor calcidiol via the hydroxylating activity of CYP27B1, an enzyme expressed in placenta and kidney. Substantial evidence has implicated calcitriol in blood pressure regulation [13,14,15,16]. Accordingly, low VD levels in serum of pregnant women have been associated with higher risk of preeclampsia and other hypertensive disorders [17,18]. At the same time, VD supplementation is known to decrease the incidence of these pathologies [19]. Many mechanisms are thought to be involved in these processes, including the potent anti-inflammatory effect of calcitriol as well as its ability to repress renin transcription, preventing in this manner ANG II accumulation and the consequent rise in blood pressure [13,20,21].

On the other hand, several studies have associated VD-deficiency with increased odds of urinary tract infections (UTI) [22,23,24], highlighting hypovitaminosis D as a potential modifiable risk factor for bacterial vaginosis among the general population including pregnant women, and emphasizing VD supplementation as a prevention strategy for UTIs [25,26]. This epidemiological data reflect the ability of calcitriol to exert microbicidal activity, which is achieved by transcriptionally inducing antimicrobial peptides expression. Among these peptides, urinary and plasma cathelicidin levels are notably increased among UTI-affected patients [27]. Cathelicidin may be produced by immune cells, epithelial cells of the urothelium, as well as by the human placenta, where it is readily stimulated by calcitriol [28,29], conferring this hormone a major role in innate defense enhancement. Indeed, cathelicidin is involved in the protection of the urinary tract and the fetoplacental unit against bacterial infections [30,31,32]. Of particular importance is the existing evidence linking infections to the development of hypertension in pregnancy [33,34,35,36]. Regarding UTI, it has an estimated incidence of 20% during gestation and has been linked to a higher risk of preeclampsia, probably through increasing the inflammatory response and/or blood pressure [37]. Accordingly, treatment of bacteriuria in pregnancy is known to significantly reduce the incidence of hypertensive pathologies [33], corroborating a connection between infection and hypertension. 

Considering all of the above, we aimed to investigate whether there is an association between the placental production of calcitriol (as reflected by its concentration in venous cord serum) and maternal blood pressure in control pregnancies and those affected with UTI. In addition, given the beneficial role of cathelicidin in fighting UTI, we studied its relationship with newborn anthropometric parameters and gestational age as markers of neonatal outcome. Finally, since existing data has shown sexual dimorphism in placental VD metabolism and cathelicidin production [38], we analyzed our results considering the neonate sex. 

## 2. Materials and Methods

The following parameters and their associations were evaluated: (1) the concentration of calcidiol and calcitriol in umbilical serum; (2) placental tissue gene expression of cathelicidin and CYP27B1; and (3) clinical and anthropometric parameters of mothers and their newborns. 

### 2.1. Subjects and Study Design

This was a cross-sectional study including pregnant women with a diagnosis of UTI (*n* = 48) at the time of delivery (UTI group) or with a normoevolutive pregnancy (NP group, *n* = 44), who delivered a single neonate vaginally or by cesarean section. The participants were in good health prior to the inclusion in this study, as determined by routine clinical examination throughout early-mid pregnancy, and were selected during admission to the maternal facility. Women with hormonal treatment or cases with newborn or placental abnormalities were excluded. The NP-group did not present inflammatory or infectious diseases, as corroborated by negative urine culture and/or urinalysis, absence of proteinuria, or UTI symptoms. The UTI-group was integrated by patients with symptomatic UTI, as defined by the presence of dysuria and high frequency and urgency of urination; urine test strip positive for leukocytes, nitrites, and alkaline pH; and/or urinalysis suggestive of positive infection (bacteriuria). In this group, women treated with an antibiotic for UTI were included. Arterial pressure in normal ranges was an inclusion criterion for both groups given that established hypertension could act as a confounding factor in our study. Considering the neonate’s sex, the following subgroups were formed: NP-females (*n* = 24), NP-males (*n* = 20), UTI-females (*n* = 22), and UTI-males (*n* = 26). In some instances, not all samples could be evaluated for all parameters due to losses during processing.

### 2.2. Sample Collection and Quantification of VD-Metabolites in Cord Serum

After delivery, a piece of tissue was collected from the central part of the placenta and preserved in RNAlater (Sigma-Aldrich, St. Louis, MO, USA) for gene expression studies. In addition, approximately 15 mL of cord blood were withdrawn exclusively from the umbilical vein after clamping. This sample was centrifuged at 3000 rpm for 10 min at 4 °C and stored in the dark at −70 °C until analysis. 

The concentration of serum calcidiol and calcitriol in each sample was analyzed by a quantitative chemiluminescent immunoassay in the LIAISON platform (Liaison XL, DiaSorin Inc., Stillwater, MN, USA) at the central laboratory of the Instituto Nacional de Ciencias Médicas y Nutrición Salvador Zubirán.

### 2.3. Real Time PCR Amplifications

Placental tissue was homogenized in the presence of Trizol (Life Technologies, Carlsbad, CA, USA) using ceramic beads and the MagNA Lyser benchtop device (Roche Diagnostics Corp., Basel, Switzerland). RNA extraction from placental homogenates continued as indicated by the manufacturer. We used 2 µg of RNA for reverse-transcription, using the Maxima First Strand Kit (ThermoFisher Scientific, Pittsburgh, PA, USA). The obtained cDNA was amplified using the Master Mix on the LightCycler 480 (Roche), following standard cycling conditions. Results were normalized against the gene expression of the housekeeping gene glyceraldehyde-3-phosphate dehydrogenase (GAPDH). Sense and antisense oligonucleotides were: for cathelicidin (NM_004345.3), tcggatgctaacctctaccg/gtctgggtccccatccat; for GAPDH (NM_002046.3), agccacatcgctcagacac/gcccaatacgaccaaatcc; and for CYP27B1 (NM_000785.2), cttgcggactgctcactg/cgcagactacgttgttcagg. Hydrolysis probes used for cathelicidin, GAPDH, and CYP27B1 were number 85, 60, and 63, respectively, from the Roche Probe Library.

### 2.4. Statistical Analysis

Two-way analysis of variance (ANOVA) was used to calculate and compare least-square means and to identify interactions between condition (UTI) and neonate sex. Correlation analyses were carried out using Spearman’s rank correlation. *p* values < 0.05 were considered statistically significant. Analyses were performed using SigmaPlot package (SigmaPlot, RRID:SCR_003210).

## 3. Results

The clinical characteristics of mothers and their neonates are summarized in Table 1. In the UTI cohort, the newborn anthropometric parameters, Apgar scores, and gestational age at delivery were all significantly reduced as compared to NP. Bacteriuria was confirmed in UTI samples. Blood pressure and body mass index (BMI) did not significantly differ among groups (Table 1).

### 3.1. The Umbilical Cord Serum Concentrations of VD-Metabolites Were Significantly Higher in UTI Samples

**Figure 1:**
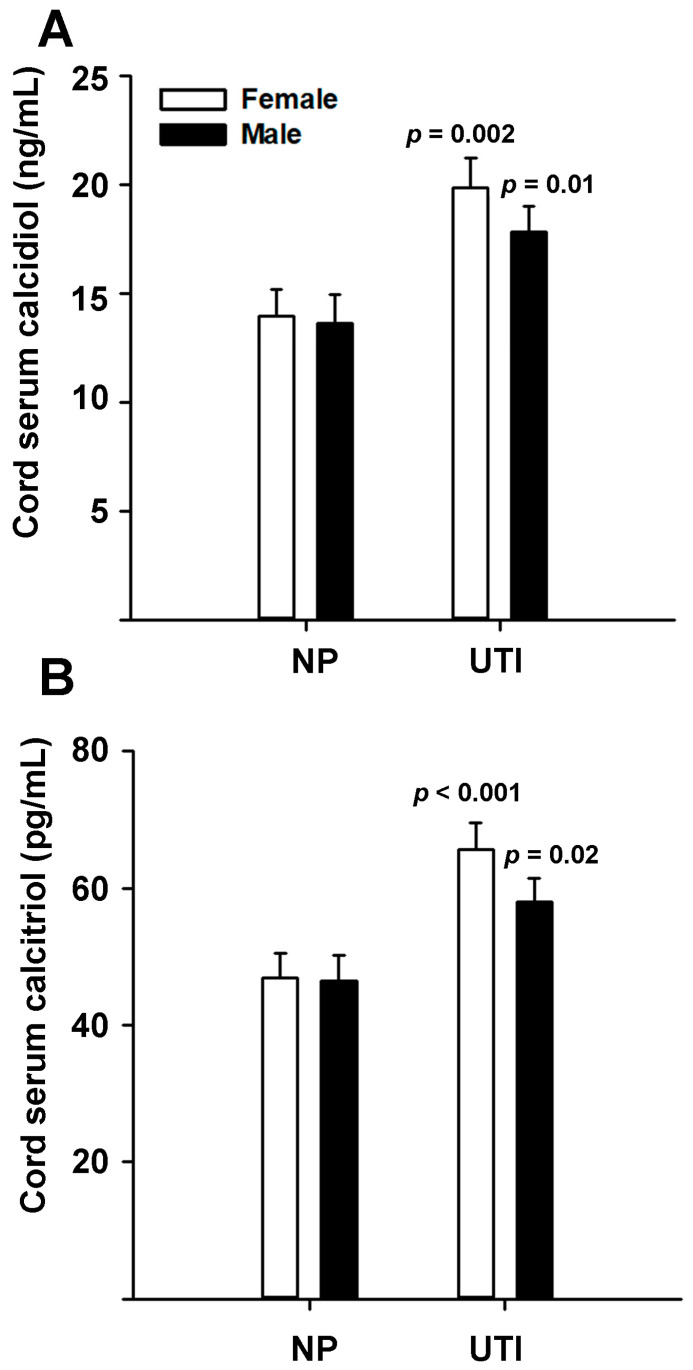
Vitamin D_3_ metabolites in cord serum from normal and UTI-affected pregnancies. Calcidiol (**A**) and calcitriol (**B**) cord serum levels were quantified in samples from normal pregnancies (NP) and with urinary tract infection (UTI). Bars represent least-square means ± SEM. The value of *p* indicates the statistical significance as compared to NP samples within same sex.

The concentration of calcidiol and calcitriol in the umbilical vein serum was significantly elevated in UTI as compared to NP groups (Figure 1A,B). Although the concentration of both VD-metabolites was higher in UTI-female samples than in UTI-males (calcidiol: 19.8 ± 1.3 ng/mL vs. 17.85 ± 1.1 ng/mL, respectively; calcitriol: 65.6 ± 4.0 pg/mL vs. 58.0 ± 3.3 pg/mL, respectively), these differences did not reach statistical significance. Notably, considering the cut-offs in VD status according to the Endocrine Society [39], calcidiol levels in both the NP group (13.8 ng/mL) and the UTI group (18.8 ng/mL) must be considered as VD-deficient (<20 ng/mL).

### 3.2. Comparative Studies of Placental Cathelicidin and CYP27B1 mRNA Expression

**Figure 2:**
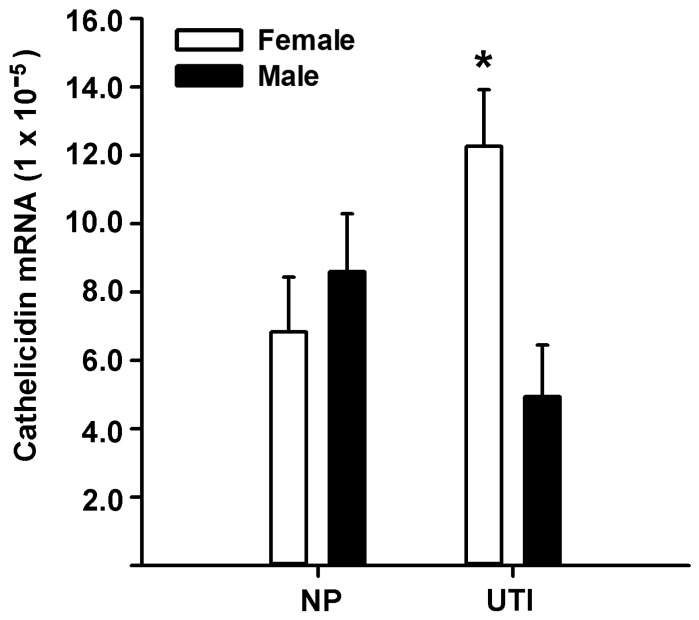
Tissue cathelicidin gene expression in placentas from normal and UTI-affected pregnancies. The relative gene expression of cathelicidin was analyzed by qPCR in placentas obtained from normal pregnancies (NP) and with urinary tract infection (UTI) and was normalized against GAPDH gene expression. Bars represent least-square means ± SEM. * *p* < 0.03 vs. NP subgroups and vs. UTI-male subgroup.

CYP27B1 gene expression was found significantly more elevated in UTI-female placentas in comparison to males from the same condition: 1.18 ± 0.21 vs. 0.60 ± 0.21, respectively (least-square means ± SEM × 10^−6^, *p* = 0.03, Student’s *t*-test). No difference in CYP27B1 gene expression was found in NP sub-groups (data not shown). Likewise, placentas associated with female neonates from UTI-affected pregnancies had higher cathelicidin gene expression compared to UTI-males (*p* < 0.05, Figure 2). Interestingly, the presence of UTI significantly elevated placental cathelicidin gene expression as compared to NP, but only in placentas obtained from female neonates. A statistically significant interaction between neonate sex and clinical condition was found (*p* = 0.006), suggesting that the sexually dimorphic difference in this trait depended on the presence of maternal infection.

### 3.3. Correlation Studies between Expression of Placental Factors, Cord Serum VD-Metabolites, and Newborn/Maternal Parameters

**Figure 3:**
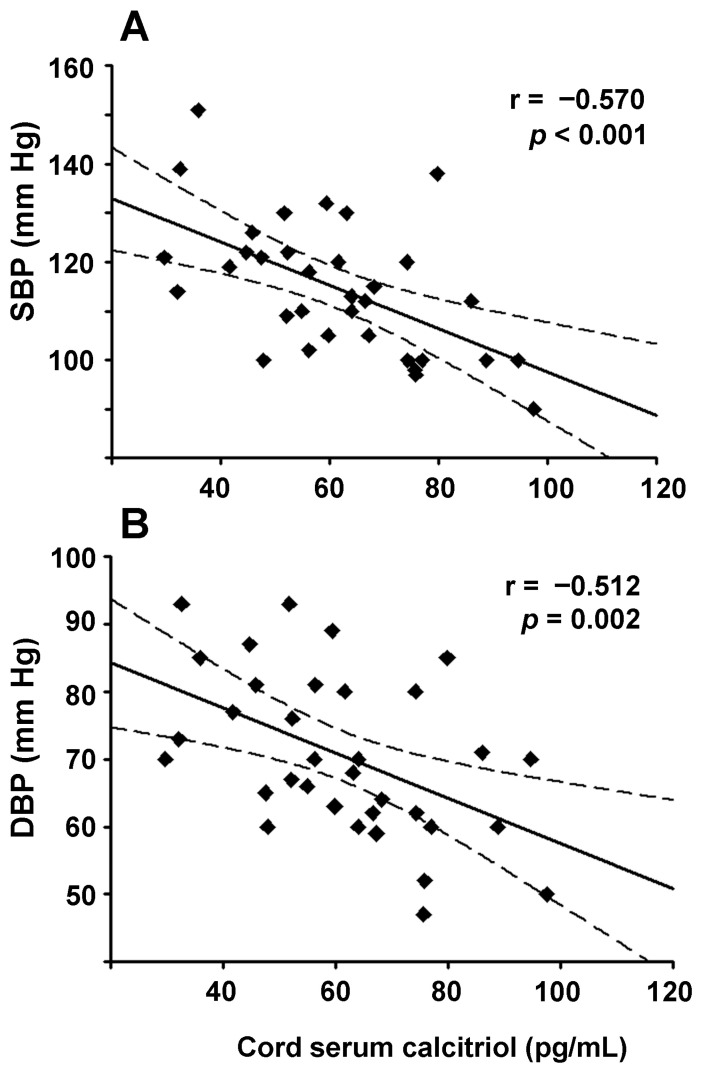
Cord serum calcitriol levels inversely correlated with maternal blood pressure in UTI. Simple linear regression plots show the associations between cord serum calcitriol and maternal systolic and diastolic blood pressure (SBP, DBP; (**A**,**B**), respectively) in samples from the cohort of urinary tract infection. Dashed lines indicate 95% confidence intervals.

In the UTI group, higher calcitriol levels in cord blood negatively correlated with maternal SBP and DBP (r = −0.57, *p* < 0.001 and r = −0.51, *p* = 0.002, respectively, Figure 3A,B). When analyzing by neonate sex, this inverse correlation was also highly significant in both male and female subgroups in the UTI condition (Table 2). Interestingly, in the NP control group, this negative association of calcitriol was observed only with SBP and only in the NP-female subgroup (r = −0.511, *p* = 0.03). Regarding calcidiol, no significant associations were found with blood pressure; however, a similar trend with borderline significance was found between calcidiol and DBP in the UTI-male subgroup (r = −0.431, *p* = 0.056).

**Figure 4:**
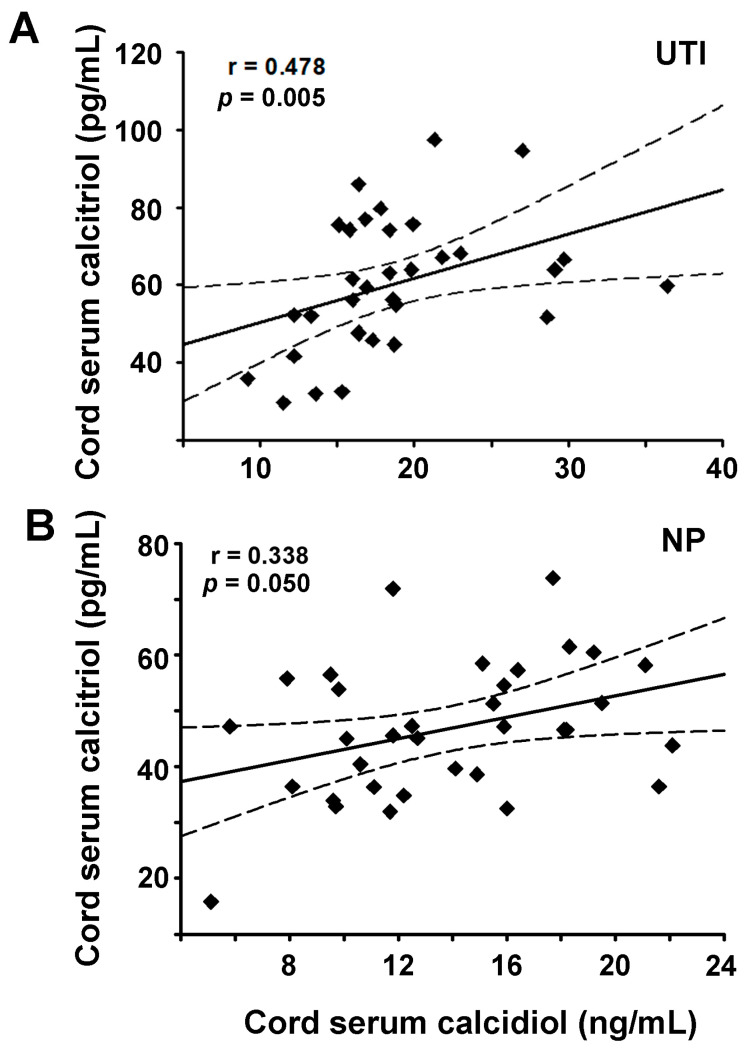
Positive correlation between cord serum levels of calcitriol and calcidiol. Simple linear regression plots depict positive correlations between calcitriol and calcidiol in cord serum samples from the cohort of urinary tract infection (UTI, **A**) and normal pregnancy (NP, **B**). Dashed lines indicate 95% confidence intervals.

Other significant associations found among studied parameters include the positive correlation between calcidiol and calcitriol in the UTI and NP groups (Figure 4A,B, Table 2), which was to be expected since calcidiol is the precursor of calcitriol. When analyzing by neonate sex, this relationship was observed only in the UTI-male subgroup, where calcitriol and calcidiol strongly correlated with high statistical significance (r = 0.703, *p* < 0.001). 

Notably, cord calcitriol negatively correlated with maternal bacteriuria but only in the UTI-female subgroup (r = −0.636, *p* = 0.01), while cathelicidin placental gene expression was positively associated with gestational age at delivery in both UTI-male and UTI-female subgroups as well as with some neonate anthropometric parameters (Table 2). On the other hand, and as expected, SBP and DBP as well as neonate anthropometric parameters, such as weight, length, and cephalic perimeter, highly correlated between them (Table 2). Intriguingly, SBP positively correlated with BMI in NP and UTI subgroups only if the neonate was female (Table 2).

## 4. Discussion

Even though hypertension in pregnancy is one of the leading causes of maternal mortality and perinatal morbidity, the triggering factors and mechanisms involved in this condition are not yet fully elucidated. Conditions, such as alterations in trophoblast invasion, placental ischemia, endothelial dysfunction, and increased production of placental vasoconstrictor factors, are among those known to be involved in pregnancy-induced hypertension and preeclampsia [40,41]. However, additional mechanisms may participate in infection-related hypertension, including systemic inflammation, which is mediated by exacerbated production of inflammatory cytokines, as occurs in UTI [37,42,43]. While these immune mediators are produced to defend against invading microbes, an exacerbated response may jeopardize pregnancy [44]. Hence, the placenta has evolved regulatory feed-back strategies, such as the production of anti-inflammatory factors like calcitriol. In fact, inflammatory cytokines and/or LPS upregulate placental CYP27B1 expression, raising calcitriol production [45,46], which in turn decreases inflammation and induces antimicrobial peptides [29,32,47]. In the present study, we provide evidence supporting that placental calcitriol may also be involved in lowering maternal blood pressure in the presence of an UTI, helping to maintain normal ranges. Indeed, under this infectious condition, both SBP and DBP negatively correlated with calcitriol levels in venous cord serum with high coefficients of correlation and statistical significance. The fact that we observed this negative correlation mainly in the UTI group may relate to the significantly higher levels of VD metabolites found in all UTI cord serum samples compared to controls. However, an inverse and significant correlation between calcitriol and maternal SBP of female-carrying mothers was also found in the NP-female subgroup, suggesting a sex-dependent trend unrelated to the clinical condition. Although correlation does not imply causation, our results, together with the well-known ability of placental calcitriol to reach the maternal circulation [10,11,12] and act as a negative endocrine regulator of RAS [48], strongly support a role of placental calcitriol in the control of maternal blood pressure. In agreement with our observations, low maternal calcitriol serum levels in a cohort of infected women have been associated with pregnancy-induced hypertension and group B Streptococcus colonization [49]. Moreover, calcitriol is known to exert a potent vasodilator effect due to the presence of the vitamin D receptor in blood vessels, playing an essential role in endothelial cell function and blood pressure control [50,51]. In addition, calcitriol inhibits parathyroid hormone (PTH) secretion, which may help prevent the PTH-dependent increase in blood pressure [16,52]. 

Interestingly, calcidiol serum concentration has been inversely associated with cardiovascular mortality in women but not in men, further suggesting an interplay between sex hormones and cardiometabolic health [53]. Indeed, sexual dimorphism has been described in the association of serum VD deficiency and increased risk for cardiometabolic disorders [54].

Even though we found higher calcidiol and calcitriol levels in UTI-female cord samples than in males, this difference did not reach statistical significance, probably due to the small size of our subgroups. However, our data showed higher placental CYP27B1 and cathelicidin gene expression in UTI-female placentas in a similar manner as described previously in healthy placentas associated with female fetuses [38]. In the last report, male newborns also had lower umbilical cord cathelicidin serum levels, attributed to diminished placental cathelicidin expression secondary to decreased calcitriol bioavailability, resulting from a testosterone-dependent inhibition of CYP27B1 gene expression [38]. Remarkably, in this study, cord serum calcitriol negatively correlated with maternal bacteriuria only in the UTI-female subgroup, further supporting a sex-dependent differential VD regulation of the immune response and suggesting that placental production of calcitriol may help to attenuate maternal UTI. In agreement with our assumption, in a randomized controlled trial, the supplementation with VD significantly prevented UTI [26]. At the same time, serum calcidiol levels less than 20 ng/mL in pregnant subjects have been shown to represent the only factor associated with UTI [23]. Furthermore, maternal serum calcidiol levels have been correlated with an increased capacity to inhibit uropathogenic *Escherichia coli* growth in vitro [55], which is in line with the ability of VD to augment cathelicidin expression in bladder tissue of *E. coli*-infected women [56].

On the other hand, in our study, cathelicidin gene expression was positively and significantly associated with neonate birth measures; for instance, with birth weight in both NP and UTI groups independently of the neonate’s sex. Additionally, in UTI, cathelicidin expression was associated with gestational age in both male and female UTI subgroups, suggesting that a higher expression of this antimicrobial peptide relates to fewer preterm births under an infectious threat. Overall, these results link placental cathelicidin to a better neonatal outcome and suggest that this antimicrobial peptide influences prenatal physical development particularly in the presence of maternal UTI. 

Various clinical and epidemiological studies have suggested a correlation between VD-deficiency and high blood pressure in different clinical and physiological settings [13,14,15,18,19,20,48,49]; however, this is the first study showing an inverse correlation between umbilical venous serum calcitriol and both SBP and DBP of pregnant subjects with UTI. Interestingly, significantly lower SBP and DBP have been reported among children and adolescents with higher cord-calcidiol levels at birth [21,57]. In contrast, maternal VD deficiency has been associated with increased susceptibility to hypertension in the offspring due to impaired endothelial relaxation of the large vessels [58]. Altogether, these evidences strongly suggest a VD-dependent intrauterine programming of the offspring’s blood pressure.

Herein, we specifically quantified VD metabolites in samples from the umbilical vein so that it would reflect placental biosynthesis. Notably, we detected VD-deficiency in all our cord serum samples, probably due to maternal deficiency, as shown elsewhere [59]. However, it should be noted that calcidiol levels in cord serum have been reported on average 25% reduced as compared to maternal serum levels [60]. Unfortunately, a high prevalence of maternal VD deficiency has been reported worldwide [61,62] and has been associated with overall adverse consequences for the mother and offspring [63]. One important limitation of the present study is that all the analyzed metabolites are mainly from fetal origin (i.e., venous umbilical cord serum). Indeed, we did not consider maternal VD metabolites. It would be interesting to measure their levels in peripheral maternal blood to visualize an integrated picture of the maternal–placental–fetal VD metabolism in the context of UTI and sexual dimorphism.

It is now well established that by activating toll-like receptors, bacterial products trigger CYP27B1 expression, which in turn increases endogenous calcitriol production and ultimately, bacterial killing [64]. Accordingly, in our study, maternal UTI significantly increased cord-calcitriol levels. However, and intriguingly, UTI also increased cord-calcidiol levels. We believe that this may be explained by the presence of binding sites for the inflammation-related transcription factor NF-kB in the CYP2R1 gene, which encodes the main VD 25-hydroxylase involved in calcidiol synthesis, the expression of which is robustly associated with this metabolite serum levels [65]. Moreover, in a study conducted in germ-free mice, hepatic cyp2r1 gene expression increased after microbial transplantation, while that of renal cyp24a1, encoding for the enzyme that catabolizes calcidiol and calcitriol, was inhibited [66]. These processes resulted in a significant increase in calcidiol serum levels. Interestingly, the authors showed that females produced more calcidiol than males, corroborating a sex-dependent VD-metabolism [66]. Further studies are needed to investigate more in depth a sexually dimorphic signature of the VD-endocrine system.

Our results warrant future research, including prospective longitudinal studies, to evaluate changes in maternal VD metabolites and immune markers along the first, second, and third trimesters of pregnancy and to relate these to the risk for hypertension and/or UTI development in the gestating mother.

## 5. Conclusions

Our findings strongly suggest that placental calcitriol biosynthesis is tightly linked to maternal blood pressure regulation and to the strength of the immune response in a sexually dimorphic manner. Specifically, the data presented herein reflect a more robust cathelicidin-dependent immune response at the fetoplacental unit of female-carrying pregnancies. We strongly recommend VD-assessment as part of routine care during pregnancy and VD-supplementation and/or solar exposure in the case of hypovitaminosis D, as an easy way to prevent UTI and high blood pressure.

## Figures and Tables

**Table 1:** Clinical data of mothers and newborns.

Clinical Data	NP-Female	NP-Male	UTI-Female	UTI-Male	NP Total vs. UTI Total
	*n* = 24	*n* = 20	*n* = 22	*n* = 26	(*p*)
**Maternal parameters:**					
Pregestational BMI (kg/m^2^)	24.7 ± 0.9	24.4 ± 0.9	24.8 ± 1.0	25.1 ± 0.8	ns
Gestational age (weeks)	38.6 ± 0.6	38.6 ± 0.7	**35.9 ± 0.7 ^b^**	**36.3 ± 0.6 ^b^**	**<0.001**
SBP (mm Hg)	115 ± 2	111 ± 3	115 ± 3	114 ± 2	ns
DBP (mm Hg)	72 ± 2	66 ± 2	70 ± 2	70 ± 2	ns
Urinalysis (pH)	6.0 ± 0.1	6.0 ± 0.2	**6.5 ± 0.1 ^b^**	**6.5 ± 0.1 ^b^**	**0.005**
Urinalysis (Bacteria U/µL)	1.7 ± 12.6	1.0 ± 13.8	**68.5 ± 14.6 ^a,b^**	**23.7 ± 12.6 ^b^**	**0.001**
**Newborn data**:					
Weight (g)	3009 ± 139	3166 ± 149	**2481 ± 153 ^b^**	**2697 ± 133 ^b^**	**<0.001**
Length (cm)	49.5 ± 0.8	50.7 ± 0.9	**46.6 ± 0.9 ^b^**	**47.3 ± 0.8 ^b^**	**0.001**
Cephalic perimeter (cm)	34.1 ± 0.4	34.2 ± 0.5	**31.7 ± 0.5 ^b^**	32.9 ± 0.4	**<0.001**
Apgar score (1 min)	8.0 ± 0.2	7.9 ± 0.2	7.4 ± 0.2	7.4 ± 0.2	**0.016**
Apgar score (5 min)	9.0 ± 0.0	9.0 ± 0.0	**8.8 ± 0.0 ^b^**	8.9 ± 0.0	**0.007**

Least-square means ± standard error of the mean (SEM). Bold font depicts statistical significance. BMI, body mass index; DBP, diastolic blood pressure; SBP, systolic blood pressure; ns, non-significant; NP, normal pregnancy; UTI, urinary tract infection. ^a^ = *p* < 0.05 vs. opposite sex within UTI; ^b^ = *p* < 0.05 vs. same sex in NP.

**Table 2:** Correlations between parameters with at least one significant result.

		Subgroups								Groups			
Correlated		NP-Female		NP-Male		UTI-Female		UTI-Male		NP-Total		UTI-Total	
Factors		r	*p*	r	*p*	r	*p*	r	*p*	r	*p*	r	*p*
Cal	SBP	**−0.511**	**0.03**	0.014	0.952	**−0.565**	**0.03**	**−0.584**	**0.006**	−0.157	0.381	**−0.57**	**<0.001**
Cal	DBP	−0.256	0.316	−0.094	0.721	**−0.551**	**0.03**	**−0.534**	**0.01**	−0.125	0.485	**−0.512**	**0.002**
Cal	UB	−0.136	0.681	−0.203	0.658	**−0.636**	**0.01**	0.346	0.881	−0.020	0.935	−0.241	0.168
Cal	calcidiol	0.418	0.082	0.232	0.378	0.011	0.964	**0.703**	**<0.001**	**0.338**	**0.05**	**0.478**	**0.005**
Cal	*LL37*	0.078	0.763	**0.559**	**0.02**	0.151	0.603	−0.163	0.498	0.289	0.1	0.076	0.666
*LL37*	NB-L	0.205	0.378	0.028	0.905	0.41	0.089	**0.544**	**0.007**	0.144	0.38	**0.423**	**0.006**
*LL37*	CP	0.181	0.44	0.055	0.82	**0.523**	**0.02**	0.255	0.237	0.101	0.53	0.201	0.207
*LL37*	NB-W	**0.494**	**0.026**	−0.017	0.94	0.45	0.058	**0.54**	**0.008**	**0.33**	**0.04**	**0.38**	**0.01**
*LL37*	GAD	0.003	0.987	0.044	0.854	**0.552**	**0.01**	**0.535**	**0.008**	0.051	0.756	**0.428**	**0.005**
*LL37*	Apgar1	0.038	0.866	−0.018	0.94	0.352	0.148	0.41	0.057	0.015	0.926	**0.331**	**0.03**
NB-W	NB-L	**0.581**	**0.003**	**0.749**	**<0.001**	**0.866**	**<0.001**	**0.939**	**<0.001**	**0.63**	**<0.001**	**0.916**	**<0.001**
NB-W	CP	**0.411**	**0.05**	0.232	0.417	**0.716**	**<0.001**	**0.662**	**<0.001**	**0.349**	**0.023**	**0.796**	**<0.001**
NB-W	GAD	0.312	0.145	0.425	0.060	**0.882**	**<0.001**	**0.82**	**<0.001**	**0.35**	**0.02**	**0.846**	**<0.001**
SBP	DBP	**0.743**	**<0.001**	**0.757**	**<0.001**	**0.784**	**<0.001**	**0.774**	**<0.001**	**0.727**	**<0.001**	**0.774**	**<0.001**
SBP	BMI	**0.532**	**0.009**	0.196	0.403	**0.522**	**0.02**	0.105	0.615	**0.384**	**0.01**	**0.302**	**0.04**

BMI, body mass index; Cal, calcitriol; CP, cephalic perimeter; GAD, gestational age at delivery; LL37, cathelicidin; NB-W, newborn weight; NB-L, newborn length; NP, normal pregnancy; UB, urinary bacteria (bacteria per µL of maternal urine); UTI, urinary tract infection. Genes depicted in italic font indicates mRNA expression. Significant values are shown in bold font.

## Data Availability

All data supporting reported results are found within this article.
